# Antegrade Posterior Interosseous Flap for Nonhealing Wounds of the Elbow: Anatomical and Clinical Study

**DOI:** 10.1097/GOX.0000000000001959

**Published:** 2018-11-07

**Authors:** Ezequiel Ernesto Zaidenberg, Pablo Zancolli, Efrain Farias Cisneros, Aden Gunnar Miller, Rodrigo Moreno

**Affiliations:** From the * Department of Orthopaedics, Italian Hospital of Buenos Aires, Argentina; † Kleinert-Kutz Instiute for Hand and Microsurgery, Louisville, Ky.; ‡ Universidad de Favaloro, Buenos Aires, Argentina; § Hand Surgery and Microsurgery Department, National Institute of Rehabilitation, Mexico City, Mexico.

## Abstract

**Background:**

The posterior interosseous artery (PIA) flap has been widely reported to cover defects at the dorsal aspect of the hand. However, the use of this flap to cover elbow defects has been rarely reported. The purpose of this study was to analyze the anatomical feasibility of the PIA flap to cover elbow soft-tissue defects and, additionally, to review the clinical outcomes of patients treated with this flap.

**Methods:**

An anatomical study was performed on 14 cadaveric specimens to assess the number of PIA perforators at the distal third of the forearm, along with the distance of the perforators from the ulnar styloid. Additionally, the pedicle distance from the pivot point to the lateral epicondyle was recorded. A clinical study in 4 patients with elbow soft-tissue defects treated with the antegrade PIA was also performed to assess viability and clinical outcomes.

**Results:**

A mean of 3 perforators (range, 2–4) of the PIA were found in the distal third of the forearm. The pedicle distance from the pivot point to the lateral epicondyle was 10 cm (range, 8–11.5 cm). In the clinical study, all cases treated with the antegrade PIA flap showed satisfactory outcomes without loss of the flap or significant partial necrosis.

**Conclusion:**

In this limited series, the antegrade PIA flap has shown to be a reliable and effective alternative for treatment of soft-tissue defects at the elbow. The PIA perforators in the distal forearm and the pedicle length allow the flap to easily reach the elbow.

## INTRODUCTION

There are many causes for posterior elbow defects, including trauma, infection, wound dehiscence, burns, radiation, decubitus ulceration, chronic inflammation, and bursa excision. Certain elbow dorsum anatomical features can make the elbow prone to chronic open wounds.^1^ These features include low vascularity or a thin layer of soft tissue covering a relatively large osseous surface mostly occupied by the olecranon process and epicondyles. These structures produce higher than average stress on even healthy skin. The elbow defects need to be reconstructed with healthy, resistant, elastic, and mobile tissue. Several types of flaps and free tissue transfers have been used to reconstruct these defects.^2^ Anconeus, brachioradialis, latissimus dorsi, and flexor carpi ulnaris muscle flaps as well as radial forearm and lateral arm fasciocutaneous flaps are some of the more commonly reported flaps for reconstruction. However, each of these flaps is associated with potential donor-site morbidity or limited coverage.^3–5^

**Fig. 1.**
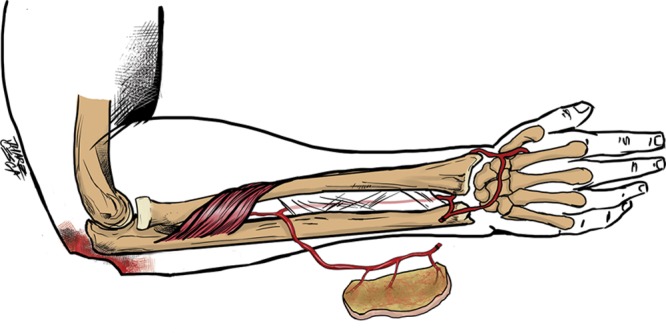
Illustration showing the proximally based (antegrade) PIA flap.

The posterior interosseous artery (PIA) fasciocutaneous flap has been described as versatile, technically easy, and is associated with a low complication rate.^6,7^ The flap can be designed as either a distal-based flap used to cover defects in the wrist or in the hand,^8–11^ or as a proximal-based flap used to cover proximal forearm defects and defects in the elbow area (Fig. 1); however, the anatomy of the distal perforators of the PIA needed for this flap has not been well described in the literature and is limited primarily to case reports describing the use of the PIA flap to cover elbow defects.^12,13^

The purpose of this study was to analyze the anatomical feasibility of the PIA fasciocutaneous flap to cover elbow defects and to assess the outcome of a case series of patients treated with the PIA antegrade flap.

## MATERIALS AND METHODS

To determine if the PIA flap routinely has sufficient number of perforators in the distal third of the arm for routine coverage of elbow defects an anatomical study was performed. Fourteen frozen cadaveric upper limbs from both sexes were used. After preparation and cannulation of the axillary artery, red latex solution was injected into each specimen. The cannulas were sealed, and the cadavers were preserved using a mixture of formaldehyde and phenolic acid according to the Cozzi technique, which allowed the injected latex to solidify evenly.^14^ In all the specimens, a dissection of the dorsal arteries of the forearm was carried out under 3.5× magnification. The number of PIA perforators at the distal third of the forearm was documented. Subsequently, the pedicle distance from the pivot point (origin of the PIA from the interosseous trunk) to the humerus lateral epicondyle was measured.

A retrospective clinical study was done with the approval of the institutional review board. A consecutive series of patients with soft-tissue defects around the elbow, treated with the antegrade PIA flap between 2011 and 2016 were analyzed. The primary indication was a defect on the posterior aspect of the elbow. However, the flap can also be used to cover small defects on the anterior, medial, and lateral aspects of the elbow.^10,12^ The patients’ demographic data can be seen in Table 1. Contraindications for this flap were severe soft-tissue injury to the posterior aspect of the forearm, history of wrist surgery through a dorsal approach or defects larger than 40 cm^2^.^15^ In all cases, the viability and size of the flap were assessed. Complications such as flap necrosis or infections were also documented.

**Table 1.**
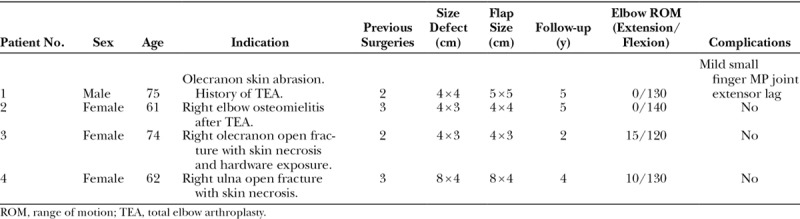
Clinical Characteristics of Case Series

### Surgical Technique

**Fig. 2.**
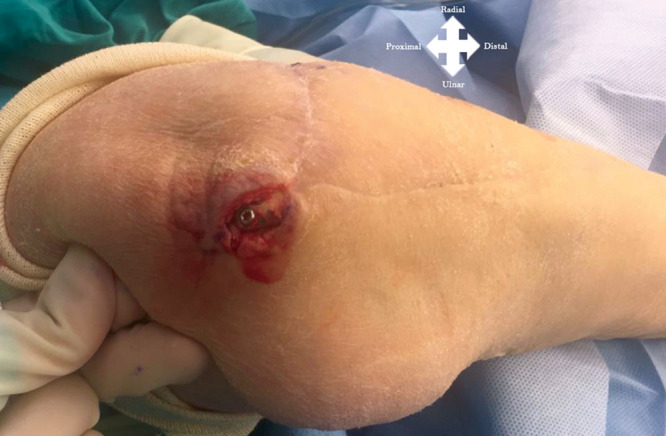
Case example. Preoperative photograph of the posterior aspect of the elbow showing the soft-tissue defect with exposure of the olecranon plate.

**Fig. 3.**
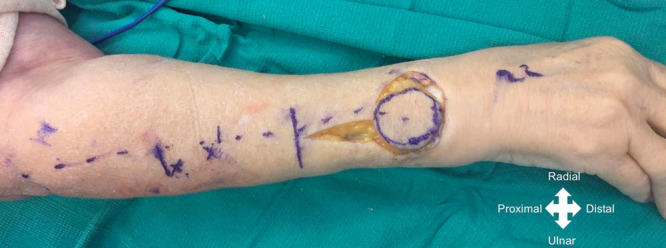
Case example. Intraoperative photograph showing the skin paddle marked distally along the axis line of the flap (from lateral epicondyle to distal radioulnar joint).

**Fig. 4.**
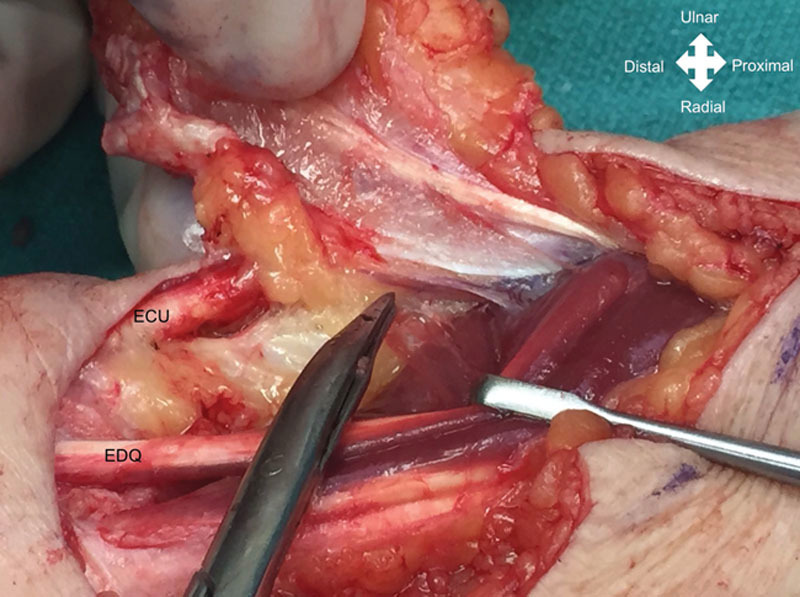
Case example. Intraoperative dissection of the PIA pedicle in the distal third of the forearm between the EDQ and ECU. EDQ, extensot digiti quinti.

All surgeries were performed by the senior author. In all patients, a regional axillary or brachial plexus block was performed, and a pneumatic tourniquet cuff was insufflated without exsanguination of the limb. The PIA flap axis was centered on a line between the lateral epicondyle to the distal radioulnar joint within the mid-forearm, between the radial and ulna bones (Figs. 2, 3). Septocutaneous perforators were identified along the intermuscular septum between the extensor carpi ulnaris (ECU) and the extensor digiti minimi (EDM; Fig. 4).

**Fig. 5.**
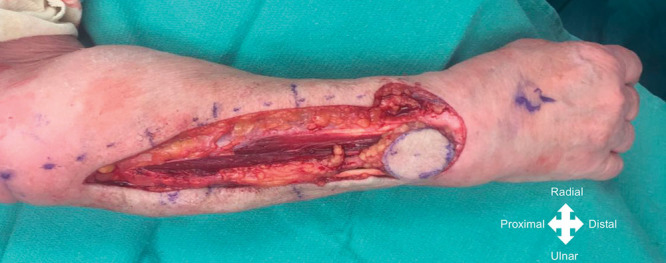
Case example. Proximal dissection of the PIA pedicle until the pivot point (origin of the PIA in the interosseous trunk).

The procedure for raising an antegrade PIA flap is somewhat different from that of a retrograde PIA flap used for coverage of hand defects. First, the perforators were outlined with a Doppler and marked on the skin. The skin paddle was positioned distally in the forearm so that it can be rotated to cover the elbow. The point of rotation is proximal at the origin of the PIA from the interosseous trunk. The distance from this point to the proximal extent of the elbow defect was measured and imposed distally on the path of the pedicle on the forearm between the fifth and the sixth extensor compartments. The skin paddle was marked distally along this line and can be raised with some extra fascia. As described by Gupta et al.,^16^ proximal to the proximal extent of the skin paddle, the fascia over the EDM and the ECU are divided longitudinally, preserving the strip of fascia between the 2. The PIA pedicle resides in this strip of fascia. The distal part of the pedicle was then clipped and the skin paddle along with the pedicle dissected proximally. It is important to be very careful with the dissection as the surgeon proceeds proximally, as the terminal branches of the posterior interosseous nerve come in close contact with the pedicle. One has to dissect the pedicle carefully away from these branches, taking care not to skeletonize the pedicle. On reaching the origin of the PIA from the posterior interosseous trunk, the flap was then ready to be rotated proximally to cover the defect (Fig. 5).

**Fig. 6.**
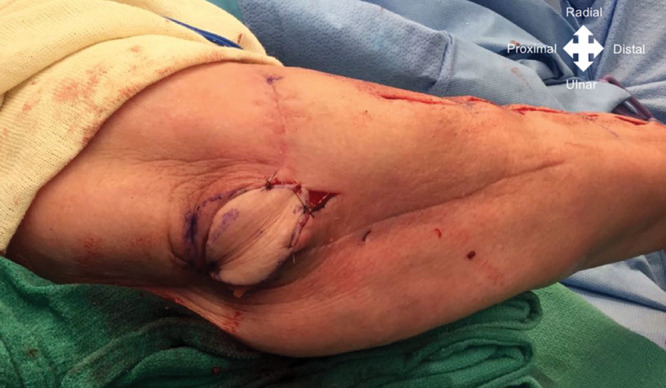
Case example. Once the dissection was completed, the PIA flap was transferred through a subcutaneous tunnel to the posterior aspect of the elbow.

The PIA is then ligated distally and tourniquet deflated to assess the vascularity of the flap. Then, the skin paddle can be transferred to the elbow defect by making a subcutaneous tunnel or by incising the skin to the defect, avoiding any pressure over the pedicle (Fig. 6).^10,15^

**Fig. 7.**
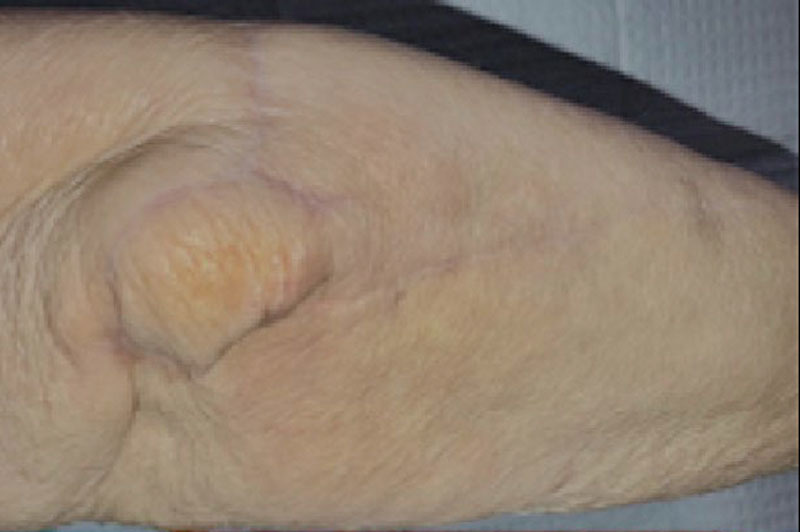
Case example. Clinical photograph at final follow-up showing complete survival of the flap with adequate cosmesis at the elbow.

**Fig. 8.**
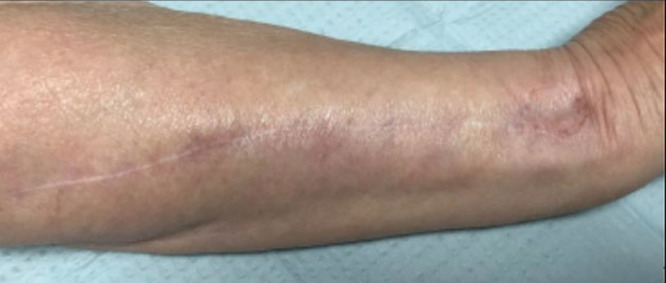
Case example. Final follow-up photograph of the donor site.

Finally, a primary closure of the donor site is possible when the width is less than 3 cm in diameter. If this size is exceeded, the authors prefer to cover the donor site with a full-thickness skin graft from the groin (Figs. 7, 8). After surgery, the upper limb was placed in an above elbow orthosis for 10 days, and rehabilitation was initiated.

## RESULTS

**Fig. 9.**
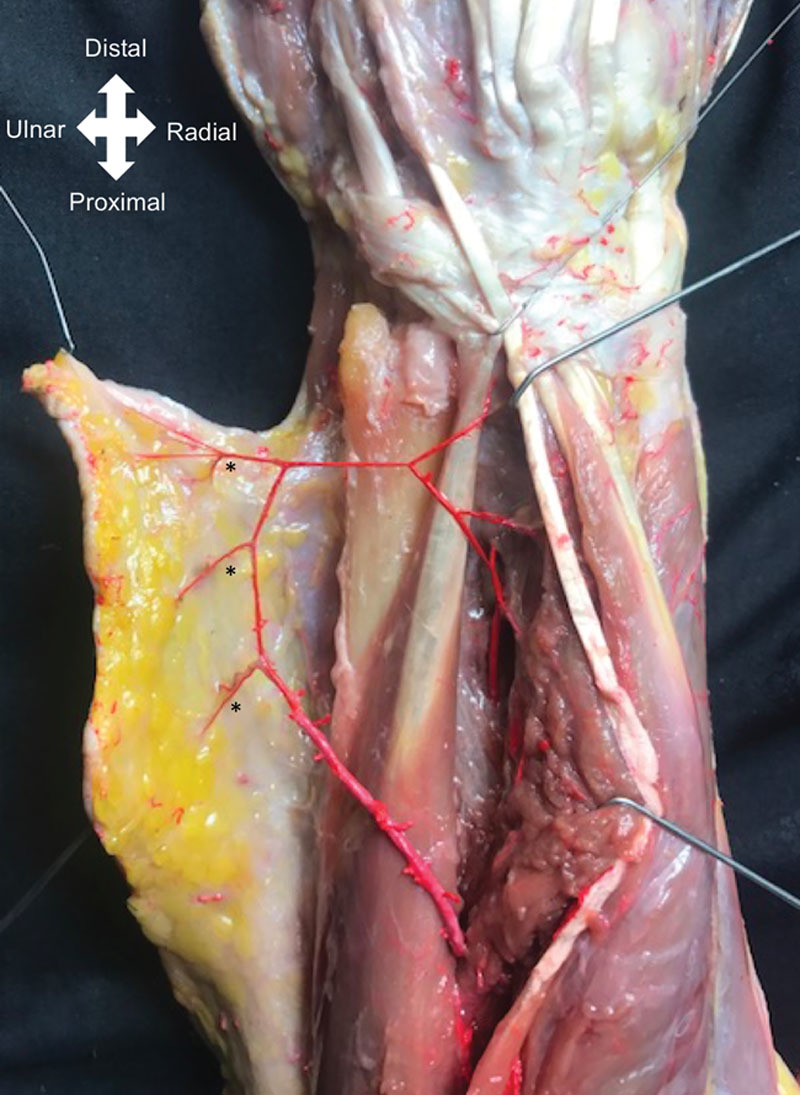
Anatomical dissection showing 3 fascio-cutaneous perforators of the PIA in the distal third forearm between the EDM and ECU.

In the anatomical study, the PIA was present in all specimens, and we found an average of 3 perforators of the PIA in the distal third of the forearm (range, 2–4; Table 2). With respect to the distal perforators location from the ulnar styloid, the first perforator was found at 3 cm (range, 2.7–4 cm), the second perforator at 5.1 cm (range, 2.8–6.8 cm), the third perforator at 7.2 cm (range, 4.5–9; Fig. 9). A fourth perforator was observed in only 5 of the 14 specimens at 7.7 cm (range, 5.9–9 cm) from the ulnar styloid. The mean external diameter of the PIA perforators was 1 mm (range, 0.5–1.6 mm). The pedicle distance from the pivot point to the lateral epicondyle was 10.3 cm (range, 8–11.5 cm), allowing the flap to reach the posterior, medial, lateral, and anterior aspect of the elbow in all 14 specimens.

**Table 2.**
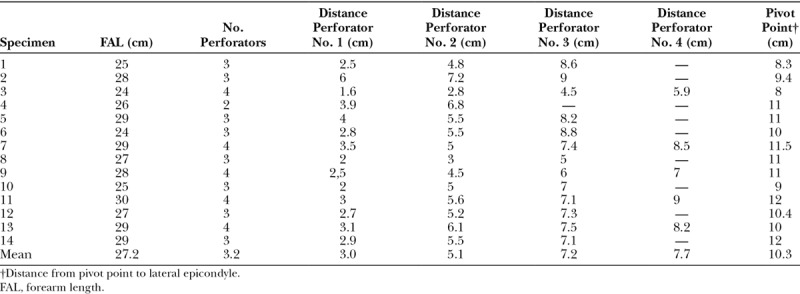
Results of the Anatomical Study

In the clinical study, 4 patients presented with soft-tissues coverage defects around the elbow that were treated with an antegrade PIA flap and had a minimum of 2 years of follow-up. The mean number of previous surgeries was 2.5 (range, 2–3). The mean follow-up was 4 years (range, 2–5 years). All antegrade PIA flaps were performed without any flap loss or significant partial necrosis. The size of the flap was based on the patient’s initial skin defect and had a mean size of 5.25 × 4 cm (range, 4–8 × 3–5). There was no need for revision of the flap, and at final follow-up, all patients were satisfied with the cosmetic outcome of the surgery and would accept to undergo the same procedure again if necessary. However, 1 patient showed a transient extensor lag of the small finger metacarpophalangeal joint, attributed to neuropraxia of the EDM nerve during proximal dissection of the pedicle, with complete recovery 6 months after surgery.

### Case Example

A 74-year-old female patient sustained an open displaced olecranon fracture with a radial head articular fracture. Initially, she was treated with open reduction and internal fixation of the olecranon as well as a radial head replacement and primary skin closure. Three weeks after the initial procedure, the patient presented a wound dehiscence of 12 cm^2^ with skin necrosis. The complication was first treated with a rotational flap to cover the defect, but the procedure failed. Subsequently, the defect was treated with a proximally based PIA flap to cover the defect of 4 × 3 cm at the posterior aspect of the elbow. The patient showed a satisfactory outcome with good cosmetic appearance.

## DISCUSSION

Soft-tissue reconstruction over the posterior aspect of the elbow remains a challenge. There are different flaps at surgeons’ disposal that can be used to cover soft-tissue defects around the elbow. The ultimate choice of flap coverage depends on several variables, including size of the wound, exposure of vital structures, comorbid conditions, and potential donor-site morbidity.^17^

Among the local muscular flaps, the anconeus is a reasonable option for coverage of the elbow. It is typically adjacent to the defect. Nonetheless, the small size and short pedicle of the anconeus limits its potential coverage area. Elhassan et al.^3^ reported good outcomes, but none of the 20 patients had any previous surgeries of the elbow. The use of the flexor carpi ulnaris and brachioradialis have also been reported with satisfactory results. However, these flaps are restricted by the small area they can cover and by their limited arc of rotation. In addition, they produce significant functional deficits in an already compromised limb.^5,16^ On the other hand, several fasciocutaneous flaps have been widely described for elbow coverage. Choudry et al.^15^ analyzed a series of 99 patients of soft-tissue coverage for posterior elbow wounds, and the most frequently utilized was the radial forearm flap. Even so, the authors explained that the main downsides to this flap include sacrificing the radial artery and donor-site morbidity and cosmesis. This flap should also be avoided in patients with a compromised ulnar artery or an incomplete palmar arch.^5^ Another common fasciocutaneous alternative is the lateral arm flap, which has the potential for less donor-site morbidity, earlier return to motion at the elbow, and preservation of major arteries. Disadvantages of this flap include the possibility of sensory deficits in the posterior brachial cutaneous nerve distribution, and an unsightly scar at the lateral aspect of the arm.^4^ In turn, the latissimus dorsi translational flap has several reports in the literature for coverage of moderate and large defects around the elbow. However, concern remains because the flap is prone to complications, as shown in certain series when the wound extended distal to the olecranon.^18^

The PIA flap has evoked great interest among upper extremity surgeons because its use avoids sacrificing a major vessel of the hand. However, reports of the use of this flap to cover elbow defects in the literature are limited.^17^ In 1986, Penteado et al.^10^ were the first to report the use of PIA flap to cover elbow defects. They treated 2 cases with satisfactory outcomes, and in the same publication, the authors described the presence of 7–14 cutaneous branches of the PIA. However, there was no mention of the mean number or location of branches at the distal third of the flap. On the other hand, Mazzer et al.^12^ reported 2 more cases treated with PIA antegrade flap to cover elbow defects with complete survival. However, they argued that this flap should not be raised too far distally in the forearm, since it may be located distally to the last “relevant” cutaneous branch of the PIA in the territory of the smaller branches, risking ischemia and necrosis. This observation is not supported by the anatomic study presented in this report, where a reliable anatomy with the presence of a mean of 3 perforators in the distal third of the forearm was observed. In the series of 4 patients reported here, the PIA flap easily reached the elbow with no evidence of congestion or necrosis. Recently Gupta et al.^16^ recommended the PIA flap and reported a satisfactory experience in 4 cases. However, no descriptions of the distal perforators were performed, and the clinical results and surgical procedure were not well described.

The present study has some limitations. First, it is retrospective in nature and is, therefore, prone to the same biases and limitations of data collection as other reviews. Second, the number of patients evaluated is relatively small. However, to our knowledge, there are no larger reports in the literature. And third, although anatomical variation was not observed in our series, it is strongly recommended that the continuity of the PIA be explored before surgery by Doppler to rule out anatomical variations.

The present study demonstrates a reliable anatomy of the distal perforators of the PIA and showed that the antegrade PIA flap is a viable option that allows to cover small-medium size elbow defects without requiring a microsurgical anastomosis or sacrifice a major forearm artery and may increase the armamentarium for soft-tissue coverage of the elbow.
